# The Influence of Tacrolimus on Cellular Morphology, Cellular Viability, Osteogenic Differentiation, and mRNA Expression within Stem Cell Spheroids

**DOI:** 10.3390/medicina60050702

**Published:** 2024-04-25

**Authors:** Won-Jong Park, Sung-Hoon Han, Hyun-Jin Lee, Ju-Hwan Kim, Hye-Jung Song, Jun-Beom Park

**Affiliations:** 1 Department of Oral and Maxillofacial Surgery, Seoul St. Mary’s Hospital, College of Medicine, The Catholic University of Korea, Seoul 06591, Republic of Korea; roll8888@naver.com; 2 Department of Orthodontics, Seoul Saint Mary’s Hospital, College of Medicine, The Catholic University of Korea, Seoul 06591, Republic of Korea; scherazade@hanmail.net; 3 Department of Periodontics, College of Medicine, The Catholic University of Korea, Seoul 06591, Republic of Korea; hyunjinlee0423@gmail.com (H.-J.L.); juhwank33@naver.com (J.-H.K.); 4 Graduate School of Clinical Dental Science, The Catholic University of Korea, Seoul 06591, Republic of Korea; hjsong55@catholic.ac.kr; 5 Dental Implantology, Graduate School of Clinical Dental Science, The Catholic University of Korea, Seoul 06591, Republic of Korea; 6 Department of Medicine, Graduate School, The Catholic University of Korea, Seoul 06591, Republic of Korea

**Keywords:** cell survival, cell differentiation, cellular spheroids, osteogenesis, stem cells, tacrolimus

## Abstract

*Background and Objectives*: Tacrolimus is a macrolide lactone compound derived from the bacterium *Streptomyces tsukubensis*, widely known as an immunosuppressant. In basic research, the effects of tacrolimus on osteogenic differentiation have been tested using mesenchymal stem cells. In this study, tacrolimus’s effects on the cellular survival and osteogenic differentiation of stem cell spheroids were investigated. *Materials and Methods*: Concave microwells were used to form stem cell spheroids in the presence of tacrolimus at final concentrations of 0 μg/mL, 0.1 μg/mL, 1 μg/mL, 10 μg/mL, and 100 μg/mL. A microscope was used to test cellular vitality qualitatively, and an assay kit based on water-soluble tetrazolium salt was used to measure cellular viability quantitatively. Alkaline phosphatase activity and an anthraquinone dye test for measuring calcium deposits were used to assess osteogenic differentiation. To assess the expression of osteogenic differentiation, a quantitative polymerase chain reaction, Western blot, and RNA sequencing were performed. *Results*: Spheroids across all concentrations maintained a relatively uniform and spherical shape. Cell viability assay indicated that tacrolimus, up to a concentration of 100 μg/mL, did not significantly impair cell viability within spheroids cultured in osteogenic media. The increase in calcium deposition, particularly at lower concentrations of tacrolimus, points toward an enhancement in osteogenic differentiation. There was an increase in COL1A1 expression across all tacrolimus concentrations, as evidenced by the elevated mean and median values, which may indicate enhanced osteogenic activity. *Conclusions*: This study showed that tacrolimus does not significantly impact the viability of stem cell spheroids in osteogenic media, even at high concentrations. It also suggests that tacrolimus may enhance osteogenic differentiation, as indicated by increased calcium deposition and COL1A1 expression. These findings advance our understanding of tacrolimus’s potential roles in tissue repair, regeneration, and stem cell-based therapeutic applications.

## 1. Introduction

Tacrolimus is a macrolide lactone compound derived from the bacterium *Streptomyces tsukubensis* [1]. Tacrolimus has played an important role in the success of solid organ transplantation worldwide, primarily as a key agent in preventing graft rejection and graft loss [2]. It is employed as part of standard therapy in conjunction with mycophenolate, mTOR inhibitors, corticosteroids, or other agents [3]. An immunosuppressant has the potential to inhibit the immune system’s assault on foreign cells, and fine-tuning the dosage of the immunosuppressant is essential to guarantee effective bone development in an allogeneic environment [4]. Tacrolimus limits T-lymphocyte activation and IL-2 transcription by inhibiting calcineurin, similar to cyclosporin, and is widely known as an immunosuppressant [5]. Tacrolimus has also been applied in dermatology, particularly for the treatment of common inflammatory skin diseases such as psoriasis and atopic eczema [6].

Existing research has shown that tacrolimus increases the osteogenic differentiation of mesenchymal stem cells [7]. Similarly, the use of tacrolimus-induced osteoblastic differentiation in vitro and can induce mineralization when delivered locally in vivo [8]. In previous research, we demonstrated that tacrolimus did not lead to statistically significant differences in the viability of gingiva-originated human mesenchymal stem cells [7]. Instead, it enhanced the osteogenic differentiation of these stem cells. A high amount of alkaline phosphatase activity, a high content of osteocalcin, and mRNAs were found in the allografts with tacrolimus. Furthermore, tacrolimus-loaded microspheres were found to increase the osteoblastic differentiation [8]. For oral soft tissue, tacrolimus has been reported to be a safe and effective medication in improving the clinical appearance of lesions, reducing pain, and diminishing the histopathological features of oral lichen planus [9]. Moreover, another study demonstrated that topical application of tacrolimus can be considered as an alternative treatment for oral erosive lichen planus resistant to steroids [10]. Additionally, it has been reported that generalized gingival enlargement can be attributed to tacrolimus-induced therapy following renal transplant [11].

To evaluate the effects of a drug on stem cells, it should be cultured in an environment that closely mimics the in vivo microenvironment. Cells require various signals, and these can influence cell proliferation and activation [12]. In the conventional two-dimensional culture system, stem cells usually grow as a monolayer, and preserving their ability for differentiation and stemness is comparatively more demanding than when they grow in multicellular environments [13,14]. Therefore, using a three-dimensional culture system would likely provide a more accurate assessment of stem cell responses to drugs. Gingiva-derived mesenchymal stem cells showed multipotent differentiation capabilities, similar or superior to mesenchymal stem cells taken from other tissues including bone marrow and adipose tissue [15]. The utility of gingiva-derived mesenchymal stem cells is heightened by their advanced differentiation capacities, which render them particularly useful in clinical applications involving tissue regeneration. Gingiva-derived stem cells may offer a number of advantages over other sources of stem cells, such as their ease of accessibility and noninvasive nature [16]. As a result, they may be an attractive option for a wide range of therapeutic applications. This present study assessed the cellular morphology, cellular viability, osteogenic differentiation, and mRNA expression of cell spheroids made of gingiva-derived stem cells in the presence of tacrolimus. Understanding how tacrolimus affects cells at a molecular level is important for the possibility of using stem cells for therapeutic purposes including tissue regeneration. Investigating the molecular effects of tacrolimus unlocks the potential for its integration into stem cell treatments and tissue regeneration, thereby extending its advantages beyond its traditional immunosuppressive functions.

## 2. Materials and Methods

### 2.1. Cell Spheroids Made from Gingiva-Derived Mesenchymal Stem Cells

The Institutional Review Board of Seoul St. Mary’s Hospital, College of Medicine, The Catholic University of Korea examined and approved the current study protocol (KC22SISE0174 was approved on 5 April 2022 and KC24SISI0005 was approved on 16 January 2024). Gingiva-derived mesenchymal stem cells were obtained, and the cells were plated on a culture dish, and any cells that did not adhere to the dish were taken out [17]. Every 2 or 3 days, we switched to a new cultural media. We created stem cell spheroids using readily available concave microwells (H389600, StemFIT 3D; MicroFIT, Seongnam, Republic of Korea). Each well was filled with a total of 1 × 10^6^ stem cells, and the cells’ reactions were then measured. We administered therapy to stem cell spheroids with tacrolimus at predetermined concentrations of 0 μg/mL, 0.1 μg/mL, 1 μg/mL, 10 μg/mL, and 100 μg/mL. On Days 1, 3, 5, and 7, we assessed the morphological traits.

### 2.2. The Assessment of Cellular Viability

In osteogenic media, we grew spheroids of stem cells. On Days 3 and 7, an assessment of the stem cell spheroids was conducted through qualitative analysis, utilizing a commercially available dual-color assay that relies on plasma membrane integrity and esterase activity (live/dead kit assay, Molecular Probes, Eugene, OR, USA) [18]. Additionally, on Days 1, 3, 5, and 7, a quantitative assessment of cellular viability was performed using an assay kit that relies on water-soluble tetrazolium salt (Cell Counting Kit-8, Dojindo, Tokyo, Japan) [19]. 

### 2.3. Levels of Alkaline Phosphatase Activity and Calcium Deposition

The assessment of osteogenic differentiation involved analyzing alkaline phosphatase activity levels and using an anthraquinone dye assay to evaluate calcium deposition [20]. Cell spheroids generated in culture wells with osteogenic medium were collected on Days 7 and 14. The assessment of alkaline phosphatase activity was conducted using a commercial kit (K412-500, BioVision, Inc., Milpitas, CA, USA). 

On Days 7 and 14, we evaluated calcium deposits using an anthraquinone dye assay to determine osteogenic differentiation [21]. After washing and fixing the stem cell spheroids, we dyed them with Alizarin Red S at room temperature for 30 min. Following the extraction, the bound dyes were quantified using cetylpyridinium chloride.

### 2.4. Total RNA Extraction and Real-Time Polymerase Chain Reaction Quantification

We processed RNA for analysis, assessing both yield and quality. Quantities falling within the absorbance range at 260 nm and 280 nm were determined using spectrophotometry (ND-2000, Thermo Fisher Scientific, Inc.). Quantitative polymerase chain reaction was employed to ascertain mRNA expression levels [22]. Based on GenBank, we created sense and antisense primers. The following are the primer sequences: RUNX2 forward 5′: AAT GAT GGT GTT GAC GCT GA–3′; reverse 5′: TTG ATA CGT GTG GGA TGT GG–3′; COL1A1 forward 5′: CCAGAAGAACTGGTACATCAGCAA–3′; and β-actin forward 5′: TGGCACCCAGCACAATGAA–3′. Applying β-actin as a housekeeping gene enabled normalization.

### 2.5. Western Blot Analysis

Cells were lysed in ice-cold radioimmunoprecipitation assay lysis and extraction buffer (Thermo Fisher Scientific, Inc., Waltham, MA, USA) according to the manufacturer’s protocols. Whole-cell lysates were quantified using the bicinchoninic acid assay (Thermo Fisher Scientific, Inc.). Protein samples were loaded and then transferred for immunoblotting. The membranes were incubated with the following primary antibodies overnight at 4 °C: anti-RUNX2, anti-collagen I, anti-RUNX2, anti-DSPP, anti-OPN, or anti-GAPDH antibody from Abcam Cambridge, UK, and Santa Cruz Biotechnology, Inc., Dallas, TX, USA. After washing, membranes were incubated with a secondary antibody from Santa Cruz Biotechnology, Inc. at room temperature.

### 2.6. RNA Isolation, Library Preparation, Sequencing and Data Analysis

Total RNA was extracted from the gingiva-derived stem cells, and RNA quality and RNA quantification were performed. Libraries were prepared from total RNA, and the isolated mRNAs were reverse transcribed into cDNA. Sequencing was performed, and quality control of raw sequencing data was performed. Pathway analysis was performed on differentially expressed genes using the Kyoto Encyclopedia of Genes and Genomes mapping tool [23].

### 2.7. Statistical Analysis

The data was presented as means and standard deviations of the experiment. Normality and equality of variance were assessed. To evaluate the impacts of concentration and time points, a two-way analysis of variance was performed. Group differences were analyzed using a one-way analysis of variance with Tukey’s post hoc test (SPSS 12 for Windows, SPSS Inc., Chicago, IL, USA). A *t*-test was used to assess group differences (SPSS 12 for Windows, SPSS Inc., Chicago, IL, USA; *p* < 0.05).

## 3. Results

### 3.1. Creation of Stem Cell Aggregates in Spheroid Form

**Figure 1:**
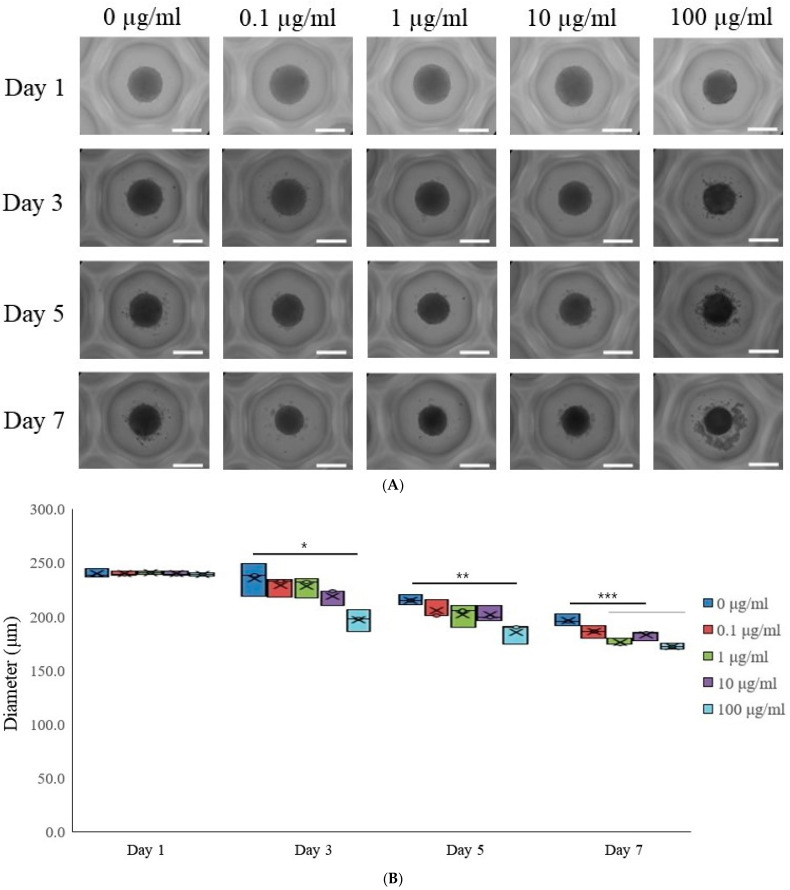
Morphological evaluation. (**A**) The morphological analysis of tacrolimus on gingiva-derived mesenchymal stem cell spheroids over 7 days. The scale bar represents 200 μm (original magnification ×200). (**B**) The morphological analysis of tacrolimus on gingiva-derived mesenchymal stem cell spheroids over 7 days. * *p* < 0.05 on day 3 compared to the time-matched unloaded group. ** *p* < 0.05 on day 5 compared to the time-matched control group. *** *p* < 0.05 on day 7 compared to the time-matched control group.

To investigate the influence of tacrolimus on gingiva-derived stem cells, each concave microwell was seeded with stem cells with a gradient of tacrolimus concentrations (0, 0.1, 1, 10, and 100 μg/mL). The spheroids’ morphological changes were documented on Days 1, 3, 5, and 7 post-tacrolimus treatment (Figure 1A). The images depict a clear progression in morphological characteristics with increasing concentration and time. On Day 1, spheroids across all concentrations maintained a relatively uniform and spherical shape. By Day 3, similar trends were seen with no significant concentration-dependent response. Days 5 and 7 show similar morphology with consistent rounded configuration.

This box plot illustrated the diameter of cell spheroids measured at different concentrations of tacrolimus (0, 0.1, 1, 10, and 100 μg/mL) across four time points (Days 1, 3, 5, and 7; Figure 1B). The central mark in each box indicates the median diameter, while the edges of the box represent the 25th and 75th percentiles. On Day 1, the diameters of spheroids across all treatment groups were comparable, with no significant variation observed. By Day 3, there was a notable decrease in the median diameter of spheroids treated with 100 μg/mL tacrolimus, as indicated by the asterisk (*), suggesting a dose-dependent response. The trend continued and became more pronounced by Day 5, with a significant reduction in spheroid diameter at 100 μg/mL (indicated by double asterisks, **). By Day 7, a significant reduction in spheroid diameter was noted at 1, 10, and 100 μg/mL (indicated by triple asterisks, ***), and the spheroid diameters in the highest concentration groups were markedly smaller than the control and lower concentration groups.

### 3.2. Assessment of Cell Viability

**Figure 2:**
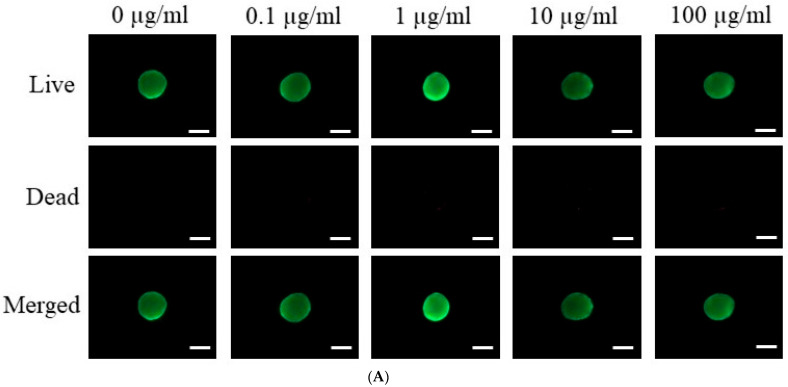

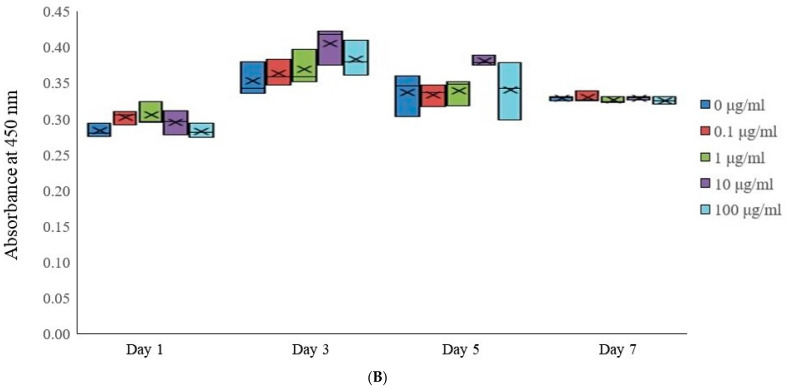
Cellular viability. (**A**) Live/Dead assay of tacrolimus-treated stem cell spheroids in osteogenic media on Day 7. The scale bar represents 200 μm (original magnification ×200). (**B**) Quantitative analysis of cell viability in tacrolimus-treated stem cell spheroids using a Cell Counting Kit-8 assay.

Figure 2A presents the results of a qualitative live/dead assay for stem cell spheroids grown in osteogenic media and treated with various concentrations of tacrolimus (0, 0.1, 1, 10, and 100 μg/mL). The assay, conducted on Day 7, employed a dual-fluorometric evaluation that differentiates live cells, which exhibit esterase activity and intact plasma membranes, from dead cells, which have compromised membrane integrity. In the live panel, spheroids across all concentrations emitted green fluorescence, suggesting robust esterase activity and intact cell membranes. The dead panel showed minimal to no red fluorescence, indicating few compromised cells across all treatment groups. The merged images corroborate these observations, with a predominance of green fluorescence and negligible red signal, implying high cell viability even at the highest concentration of tacrolimus. These results suggest that tacrolimus, up to a concentration of 100 μg/mL, does not significantly compromise the viability of stem cells within spheroids in osteogenic media after 7 days of treatment.

Cellular viability within stem cell spheroids subjected to various concentrations of tacrolimus (0, 0.1, 1, 10, and 100 μg/mL) was quantitatively assessed on Days 1, 3, 5, and 7 using the Cell Counting Kit-8 assay (Figure 2B). This assay employed a water-soluble tetrazolium salt, which is reduced by cellular dehydrogenase activities in viable cells to form a soluble formazan dye, quantifiable by measuring absorbance at 450 nm. On Day 1, spheroids across all tacrolimus concentrations exhibit similar median viability, as indicated by the comparable absorbance values. By Day 3, there was a slight variation in cell viability, with the 10 μg/mL concentration group showing an increase in mean and median absorbance. By Day 5, the 10 μg/mL concentrations indicate a more pronounced increase in absorbance, suggesting increased cellular viability. These results suggest a concentration-dependent impact of tacrolimus on the viability of cells within the spheroids, with a dose-dependent increase in cellular viability.

### 3.3. Alkaline Phosphatase Activity Levels and Calcium Deposition Extent

**Figure 3:**
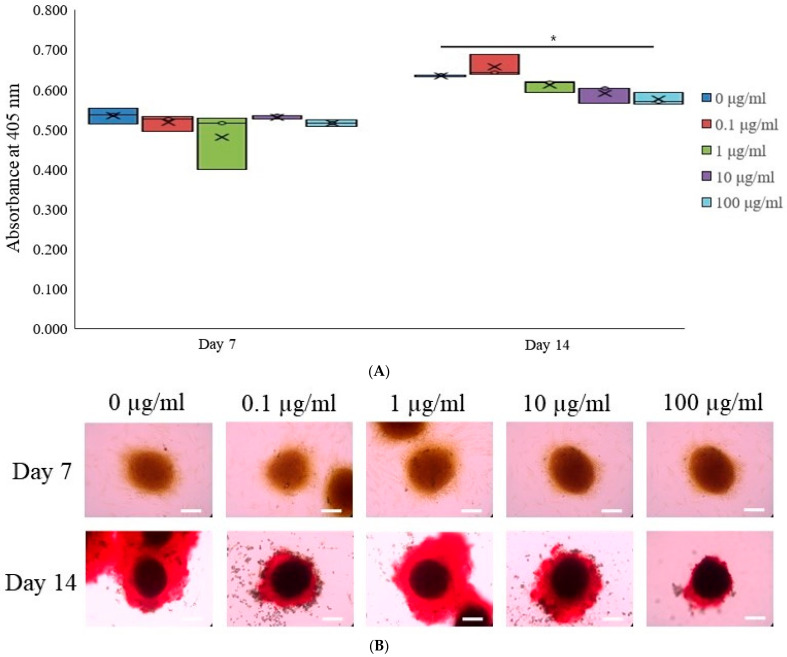

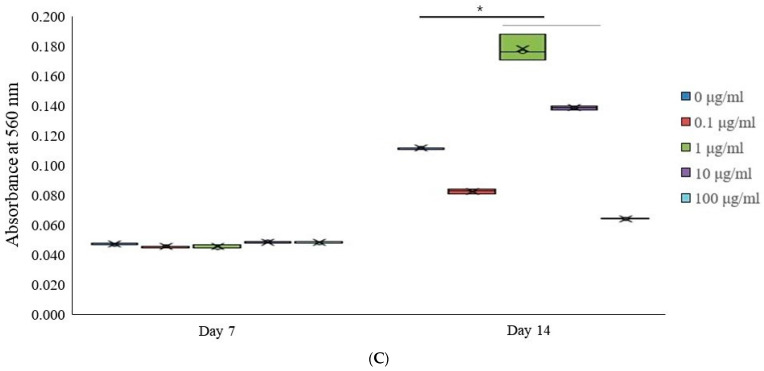
Osteogenic differentiation. (**A**) Alkaline phosphatase activity in tacrolimus-treated stem cell spheroids over 14 days. * *p* < 0.05 on day 14 compared to the time-matched unloaded group. (**B**) Evaluation of calcium deposition in tacrolimus-treated stem cell spheroids. (**C**) Quantitative analysis of calcium deposition in tacrolimus-treated stem cell spheroids. * *p* < 0.05 on day 14 compared to the time-matched unloaded group.

To evaluate osteogenic differentiation, we quantified the levels of alkaline phosphatase activity, a key early osteogenic marker, in stem cell spheroids cultured in osteogenic media. The spheroids were treated with tacrolimus at concentrations of 0, 0.1, 1, 10, and 100 μg/mL and collected on Days 7 and 14 for analysis. ALP activity was assessed using a commercial ALP assay kit (Figure 3A). On Day 7, there was a relatively uniform distribution of ALP activity across all tacrolimus concentrations, with no significant differences observed. By Day 14, however, there was a noticeable increase in ALP activity in the 1 μg/mL tacrolimus-treated group, as indicated by the asterisk, suggesting enhanced osteogenic differentiation at this concentration. These results highlight the dose-dependent effects of tacrolimus on osteogenic differentiation in stem cell spheroids, suggesting low concentrations of tacrolimus may promote osteogenic activity.

Osteogenic differentiation of stem cell spheroids was assessed using the extent of calcium deposition using Alizarin Red S staining, which binds to calcium deposits within the extracellular matrix (Figure 3B). The upper panel shows the intensity of the color, indicative of early mineral deposition on Day 7, which appeared to be similar across all concentrations. The lower panel represents spheroids on Day 14, where the staining intensity has increased and shifted to a robust red coloration, characteristic of mature calcium deposits. Notably, spheroids treated with 1 and 10 μg/mL of tacrolimus exhibited more pronounced staining, indicating greater calcium deposition and, consequently, more advanced osteogenic differentiation. These findings demonstrate a dose-dependent effect of tacrolimus on the osteogenic differentiation of stem cell spheroids on Day 14.

Calcium deposition within stem cell spheroids cultured in osteogenic media and treated with tacrolimus was quantitatively assessed on Days 7 and 14 (Figure 3C). After staining with Alizarin Red S (Sigma-Aldrich; Merck Millipore, Darmstadt, Germany), which binds to calcium deposits, the spheroids were processed to extract the bound dye. The extracted dye was quantified spectrophotometrically using cetylpyridinium chloride, with absorbance read at 560 nm, correlating with the amount of calcium-bound dye. On Day 7, there were no significant differences in calcium deposition among the different treatment groups, as indicated by the similar absorbance levels. However, by Day 14, there was a statistically significant increase in calcium deposition in the 1 and 10 μg/mL tacrolimus-treated group (indicated by the asterisk, *), as reflected by the higher mean and median absorbance. These findings indicate a concentration-dependent effect of tacrolimus on calcium deposition in stem cell spheroids.

### 3.4. Total RNA Extraction and Real-Time Polymerase Chain Reaction Quantification

**Figure 4:**
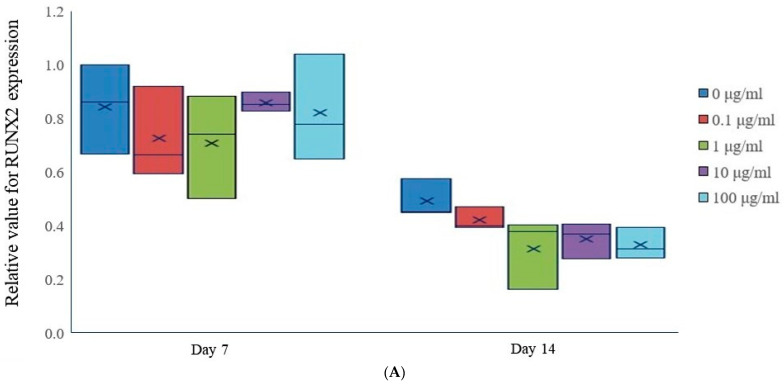

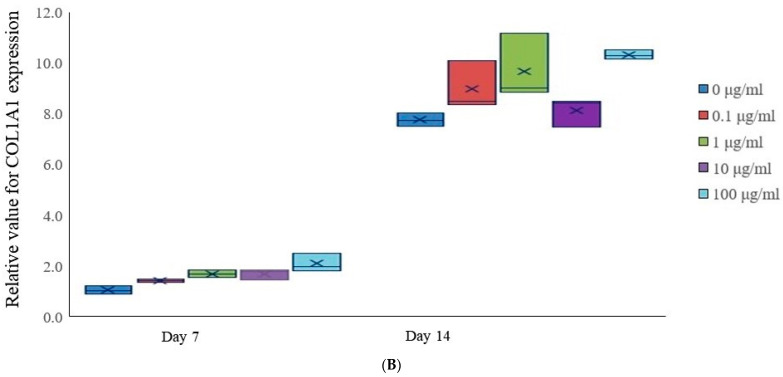
mRNA expression. (**A**) Relative RUNX2 expression in tacrolimus-treated stem cell spheroids. (**B**) Relative COL1A1 gene expression in tacrolimus-treated stem cell spheroids.

On Day 7, the median expression levels of RUNX2 were relatively similar across all treatment groups (Figure 4A). However, by Day 14, there was a decrease in RUNX2 expression across all tacrolimus concentrations, with higher concentrations of tacrolimus showing a more pronounced downregulation.

On Day 7, the expression of COL1A1 was relatively uniform across all treatment groups, with no significant differences (Figure 4B). However, by Day 14, there was an increase in COL1A1 expression across all tacrolimus concentrations, as evidenced by the elevated mean and median values, which may indicate enhanced osteogenic activity.

### 3.5. Western Blot Analysis

**Figure 5:**
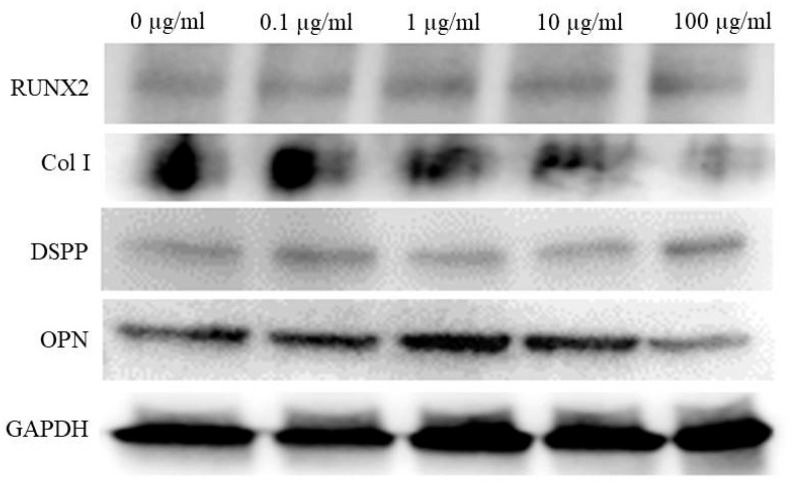
Western blot analysis of osteogenic marker expression in tacrolimus-treated cell spheroids.

The Western blot reveals expression levels of RUNX2, collagen I, DSPP, OPN, and GAPDH. Collagen I and OPN expression exhibited variations with tacrolimus treatment, providing insights into the differentiation status of the cells under different treatment conditions (Figure 5). GAPDH levels remained consistent across all samples, confirming equal loading of the proteins.

### 3.6. Data Analysis of RNA Sequencing

**Figure 6:**
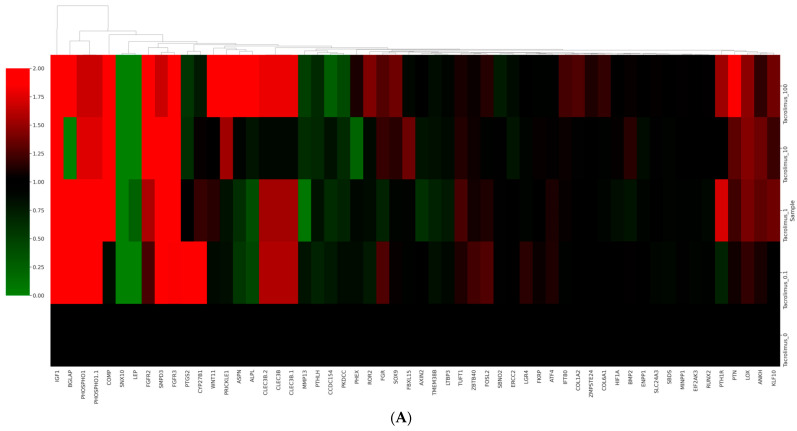

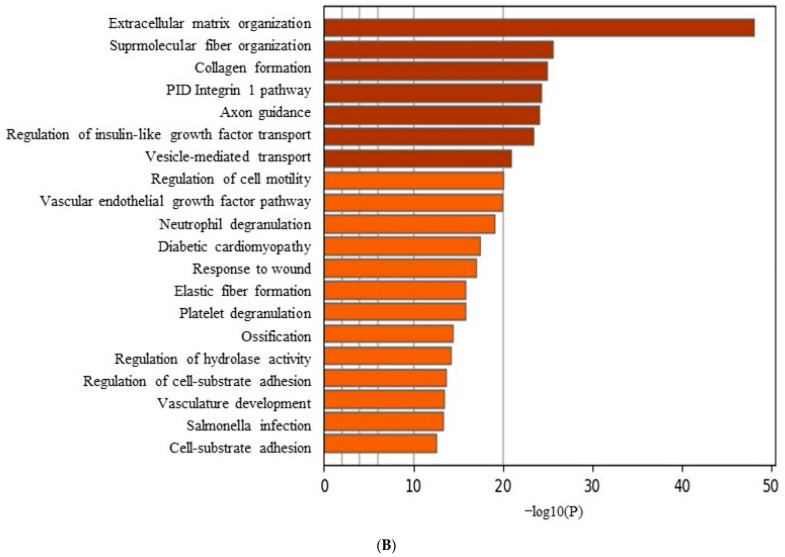

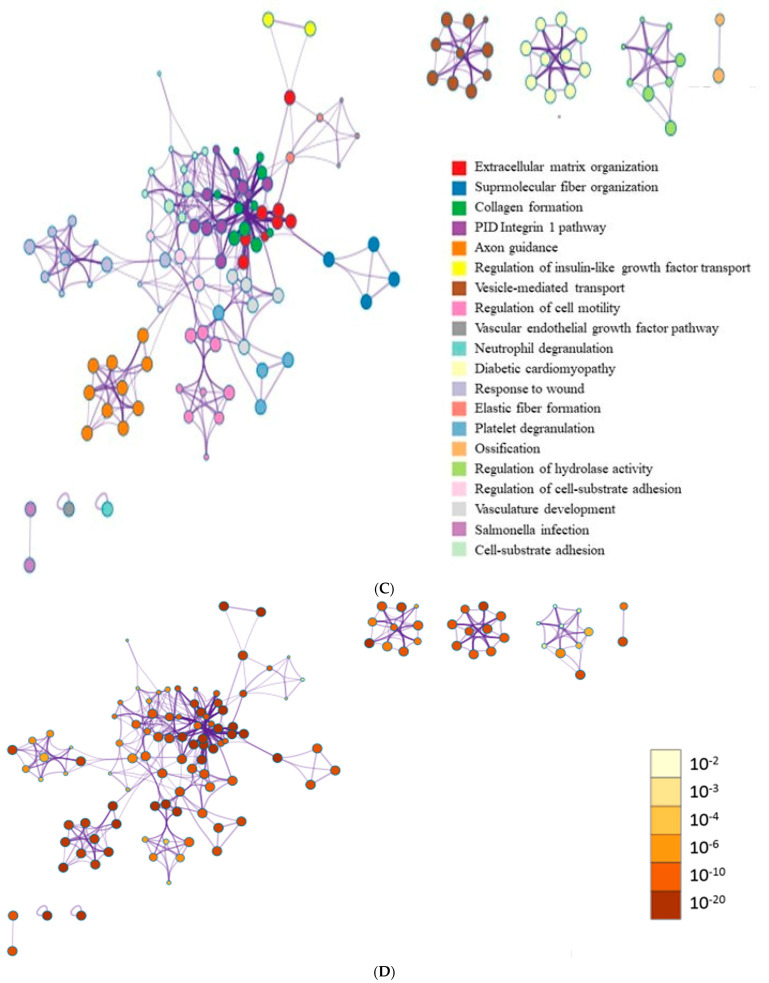

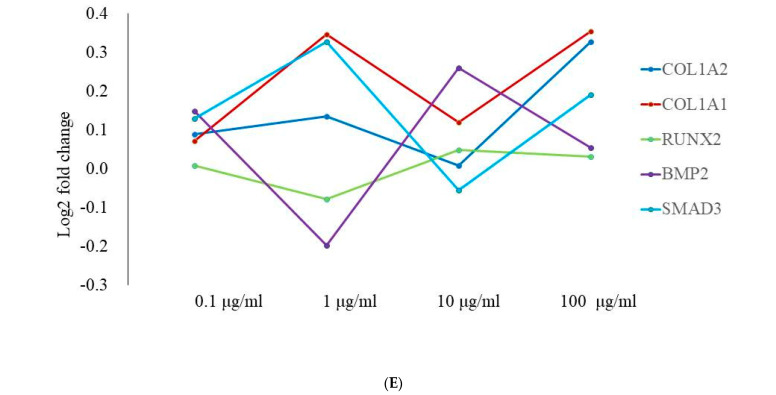
RNA sequencing. (**A**) A cluster map (a heatmap with hierarchical clustering) for osteoblast differentiation in tacrolimus-treated gingiva-derived stem cell spheroids. (**B**) Functional enrichment analysis. (**C**) Relationship between pathways (colored by cluster). (**D**) Relationship between pathways (colored by *p* value). (**E**) Gene expression profiling for regulation of osteogenic differentiation in tacrolimus-treated stem cell spheroids.

Figure 6A shows a cluster map, known as a heatmap, with hierarchical clustering for osteoblast differentiation in tacrolimus-treated stem cell spheroids. This illustrates the expression levels of multiple genes across several different conditions. Red, green, and black indicate higher expression, lower expression, and average expression, respectively. Dendrograms, the tree-like structures at the top, show the results of hierarchical clustering, which groups genes based on the similarity of their expression profiles. Further functional enrichment analysis was performed, and it was suggested that tacrolimus may influence the regulation of collagen formation and ossification (Figure 6B). The relationships between paths are shown as a network colored by clusters in Figure 6C, and the relationships between paths are colored by *p*-values (Figure 6D). Figure 6E demonstrated gene expression profiling for the regulation of osteogenic differentiation in tacrolimus-treated gingiva-derived stem cell spheroids. The line graph shows the log2 fold-change in gene expression for RUNX2, COL1A1, COL1A2, BMP2, and SMAD3 at different concentrations of tacrolimus. COL1A1 and COL1A2 expression was upregulated (i.e., increased expression relative to control) in a dose-dependent manner.

## 4. Discussion

The findings of this study demonstrate that tacrolimus influenced the cellular viability, osteogenic differentiation, and mRNA expression within stem cell spheroids. These effects were dose-dependent, with higher concentrations of tacrolimus exerting more pronounced changes. The observed alterations in gene expression suggest potential regulatory roles of tacrolimus on the osteogenic differentiation of the stem cell spheroids.

The dose-dependent morphological changes induced by tacrolimus, as evidenced by alterations in spheroid diameters, suggested the induction of differentiation pathways or potential cytotoxic effects at higher concentrations. Low doses of tacrolimus might have minimal or no detectable changes in the cellular morphology of stem cells [7]. The observed reduction in spheroid size at elevated tacrolimus levels warrants further investigation into the mechanisms of action, potentially involving apoptosis or alterations in cell proliferation rates.

Cell viability assays, including live/dead staining and Cell Counting Kit-8 analyses [24], indicated that tacrolimus, up to a concentration of 100 μg/mL, did not significantly impair cell viability within spheroids cultured in osteogenic media. These results are encouraging for the application of tacrolimus in tissue engineering, suggesting that it maintains cell survival while possibly promoting osteogenic differentiation, as inferred from the qualitative and quantitative assessments of osteogenic markers [8]. Previous study focused on adipose-derived stem cells treated with varying doses of tacrolimus. It was found that tacrolimus can cause changes in stem cell proliferation and pluripotency, which are dependent on the dosage and timing of the application [25]. Another study examined the effects of tacrolimus, alongside other immunosuppressive drugs, on mesenchymal stem cells, and this research found that short-term exposure to these drugs did not induce toxicity or apoptosis in mesenchymal stem cells [26]. Results may vary depending on the dosage. 2.4 × 10^−9^ M tacrolimus did not lead to a significant change in stem cell proliferation in the first 24 h. However, doses of 2.4 × 10^−7^ M and 2.4 × 10^−6^ M tacrolimus significantly increased the proliferation of stem cells in the first 24 h [25].

The increase in calcium deposition, particularly at lower concentrations of tacrolimus, points toward an enhancement in osteogenic differentiation [8]. This is further supported by the differential expression of osteogenic markers at the mRNA and protein levels, highlighting the potential of tacrolimus to influence osteoblast lineage commitment and maturation [27]. The quantitative polymerase chain reaction and RNA sequencing analyses provided a comprehensive overview of the molecular landscape underpinning the effects of tacrolimus on stem cell spheroids [28]. Recently, RNA sequencing has been used more widely for purposes such as extracting RNA from biological samples, enabling the study of gene expression patterns [29]. The expression profiles of osteogenic genes, including high-throughput sequencing data, offered insights into the complex regulatory networks affected by tacrolimus. The observed gene expression patterns suggest that tacrolimus may modulate pathways critical for osteogenic differentiation. Short-term exposure to tacrolimus did not induce toxicity or apoptosis in mesenchymal stem cells, but high-dose tacrolimus-induced toxicity after 7 days [30]. 

In our study, we analyzed the relative mRNA expression levels of RUNX2 and COL1A1 in stem cell spheroids cultured in osteogenic media and exposed to different concentrations of tacrolimus. RUNX2 is a key transcription factor in osteoblast differentiation and bone formation [31]. COL1A1 encodes the pro-alpha1 chains of type I collagen, a pivotal structural protein in the bone matrix and a critical marker for bone formation and osteogenic differentiation [32]. This research indicated that the expression of RUNX2 did not yield substantial outcomes related to osteogenic differentiation. Nevertheless, there was a surge in the expression of COL1A1 following the incorporation of tacrolimus. It was reported that COL1A1 was significantly involved in the osteogenic differentiation of mesenchymal stem cells [33]. COL1A1 provides a structural framework in the extracellular matrix of bone, facilitating the deposition of calcium phosphate to form bone mineral [34]. COL1A1 interacts with various signaling pathways that are essential for stem cell differentiation including the bone morphogenetic protein signaling pathway [35]. It was suggested that materials that incorporate COL1A1 can enhance the osteogenic differentiation of stem cells, promoting bone regeneration in clinical applications by mimicking the natural extracellular matrix [36,37].

There may be certain limitations to this study. This study specifically investigates the influence of tacrolimus in a controlled laboratory setting using concave microwells, which may not fully replicate the intricate in vivo environment [38]. This limitation could potentially affect how the findings are translated into practical applications. The study might not address the long-term consequences of tacrolimus exposure on stem cell spheroids [39]. Although the study examines short-term viability and differentiation, the extended effects, which are essential for clinical translation, may need further investigation. The study evaluates a range of concentrations, but the biological implications of each concentration may not be equally explored [40]. These potential limitations emphasize the necessity for further research to validate and expand upon the study’s results, particularly in more physiologically relevant models and over extended periods to confirm the safety and efficacy of tacrolimus for regenerative medicine applications.

Tacrolimus has been applied for various purposes. Tacrolimus affects the proliferation and differentiation of neural stem cells [41]. Tacrolimus has been reported to have neuroprotective effects, potentially beneficial for neural stem cells by creating a more supportive environment for their survival and differentiation [42]. The present research explored the influence of tacrolimus on the behavior of cells within stem cell spheroids. Specifically, it examined how tacrolimus affects osteogenic differentiation and modulates the expression of mRNA in these three-dimensional structures. The findings of this study highlight the need for additional research to further elucidate the precise mechanisms underlying these effects and their implications for regenerative medicine and transplantation strategies. The importance of assessing the long-term effects of tacrolimus on the viability and osteogenic differentiation of stem cell-derived spheroids cannot be overstated, particularly in light of their potential use in clinical applications. These results have significant implications for the use of tacrolimus in regenerative medicine and transplantation therapies. Understanding its effect on stem cell behavior within spheroids may aid in optimizing protocols for tissue engineering applications and enhancing the outcomes of stem cell-based transplantation therapies.

## 5. Conclusions

This study showed that tacrolimus does not significantly impact the viability of stem cell spheroids in osteogenic media, even at high concentrations. It also suggests that tacrolimus may enhance osteogenic differentiation, as indicated by increased calcium deposition and COL1A1 expression. These findings advance our understanding of tacrolimus’s potential roles in tissue repair, regeneration, and stem cell-based therapeutic applications.

## Data Availability

This article contains all of the information that was created or examined during this investigation.
